# Analysis of genetic variation in human papillomavirus type 16 E1 and E2 in women with cervical infection in Xinjiang, China

**DOI:** 10.1186/s12920-021-01120-9

**Published:** 2021-11-12

**Authors:** Luyue Wang, Fang Wang, Shaowei Fu, Chunhe Zhang, Xiangyi Zhe, Hongtao Li, Dongmei Li, Renfu Shao, Zemin Pan

**Affiliations:** 1 Department of Biochemistry and Molecular Biology, School of Medicine, Xinjiang Endemic and Ethnic Disease and Education Ministry Key Laboratory, Shihezi University, Shihezi, 832002 Xinjiang People’s Republic of China; 2 School of Science, Technology and Engineering, Genecology Research Centre, University of the Sunshine Coast, Sippy Downs, QLD 4556 Australia

**Keywords:** Cervical exfoliated cells, Human papillomavirus, E1 and E2 genes, Genetic polymorphism, Sanger sequencing

## Abstract

**Background:**

Xinjiang is one of the regions with a high incidence of cervical cancer, and the genetic variation of human papillomavirus may increase its ability to infect the human body and enhance virus-mediated immune escape ability.

**Methods:**

Sanger sequencing of the HPV16 genome from 165 samples positive for HPV16 infection and phylogenetic analysis of the E1 and E2 genes revealed the gene polymorphism of HPV16 in Xinjiang.

**Results:**

The results showed that there were 109 samples with variations in HPV16 E1, 48 sites with nucleotide variations (19 missense variations and 29 synonymous variations), and 91 samples with variations in HPV16 E2, 25 sites with nucleotide variations (20 missense variations and five synonymous variations).

**Conclusions:**

From the phylogenetic tree results, 149 samples were of the European variant and 16 samples were of the Asian variant. No African or North American/Asian variant types were found.

## Background

Cervical cancer is one of the most common malignant tumors in women worldwide. According to the 2018 global cancer burden reported in the global cancer epidemiology database, the incidence and mortality of cervical cancer rank fourth among women [1]. The occurrence and development of cervical cancer are closely related to the development of the country and the economic and health conditions. As China is the most populous of developing countries, the number of patients with cervical cancer is still growing [2]. The incidence and mortality of cervical cancer in China vary from region to region, and the central and western regions are still the key areas of cervical cancer prevention and control in China [3]. The mortality rate in the western region is slightly higher than that in the central and eastern regions, which is related to the low rate of cervical cancer screening and high HPV (Human Papillomavirus) infection rate in the less developed regions. The positive rate for HPV in Xinjiang was 14.02%, and most cases involved HPV16, 18, 52 and 53 [4].

HPV infection is a recognized leading cause of cervical cancer worldwide [5]. According to the advance phylogenetic classification of HPV, the natural genetic variation of the virus is generally divided into four major variation lineages, the A lineage: European prototype EUR(A1-A3), Asian As(A4); B lineage: African type I (AF-1); C lineage: African type II (AF-2); D lineage: Asian-American type (AA) and North American type (NA) [6]. The epidemiology of infection with HPV strains in different countries and regions varies; the rate of infection with HPV16 in women in Xinjiang is high, and most of them are European strains [7, 8]. The HPV16 genome consists of six early genes (E1, E2, E4, E5, E6, E7), two late genes (L1, L2), and an upstream regulatory region (URR). E1 and E2 are highly conserved gene sequences that regulate viral genomic replication [9]. HPV E1, one of the most conservative proteins, plays a central role in initiating HPV DNA replication. The E2 protein is a key protein in the viral life cycle, and plays an important role in transcriptional regulation, initiation of DNA replication, and viral genome segmentation [10, 11]. The structure of the HPV16 E2 protein is similar to that of a typical transcription factor, with a trans-activation domain and a carboxy-terminal DNA-binding domain separated by a variable hinge region. At the origin of DNA replication, E2 interacts with the HPV E1 replication helicase to promote cellular DNA replication [12]. HPV E2, through interaction with chromatin adapter proteins, binds the viral free genome to the host mitotic staining system during the division of infected cells, playing an important role in the segmentation of the viral genome [13]. The deletion of the E2 structure of the HPV16 virus gives rise to worse clinical consequences in patients with HPV16-positive tumors [14]. The integration of viral DNA, the destruction of E1 or E2 gene, and the loss of the inhibitory activity of the E2 protein on the early promoter are important steps in the process of malignant transformation [15–17]. The E1 and E2 proteins of HPV16 play an important role in the process of HPV infection in host cells. Therefore, studying the impact of amino acids on HPV infection is of great significance for the occurrence and development of cervical cancer.

Variations at the nucleotide sites of HPV16 E1 and E2 are associated with the development of cervical cancer [18, 19]. Therefore, analyzing the gene polymorphism of female cervical cancer in Xinjiang helps us to better understand the relationship between HPV gene variation and cervical cancer (Table 1).

**Table 1:** Information on primer sequences

Primer name	Primer sequence
E-1F	5′-AAGCACACACGTAGACATTCG-3′
E-1R	5′-ACTGCAACCACCCCCACT-3′
E-2F	5′-GAGCTGCAAAAAGGAGATTATTTGA-3′
E-2R	5′-ATCCATTCTGGCGTGTCTCC-3′
E-3F	5′-TCATGGGGAATGGTTGTGTT-3′
E-3R	5′-CAAAACATATTACAGACCCTTGCAG-3′
E-4F	5′-GGTGATTGGAAGCAAATTGTTATG-3′
E-4R	5′-TCTAGGCGCATGTGTTTCCA-3′
E-5F	5′-GCACGAGGACGAGGACAAG-3′
E-5R	5′-CCCGCATGAACTTCCCATAC-3′
E-6F	5′-GAAGCATCAGTAACTGTGGTAGAGG-3′
E-6R	5′-TGTAACAATTGCACTTTTATGTTTT-3′

F: forward primer, R: reverse primer

## Results

### Variation analysis of HPV16 E1 and E2 genes

HPV16 E1 and E2 gene variation analysis: A total of 165 DNA samples positive for HPV16 infection were sequenced, and finally 115 samples mutated in E1 and E2 were obtained. The HPV16 prototype (European prototype, GenBank accession number: NC_001526.2) was used as the standard strain for comparison. The polymorphic sites are shown in Tables 2 and 3 (End of article).

**Table 2:** HPV16 E1 gene variations and amino acid substitutions

HPV16 E1 nucleotide variation						
Variant site	AA	As	Af	Number of variation samples	Variation frequency	Amino acid change
				(n = 109)	(%)	
T98C	T	T	T	3	2.75	I33T
T105C	T	T	T	1	0.92	D35D
A117C	A	A	A	3	2.75	E39D
A189C	A	A	A	18	16.51	E63D
G195A	G	G	G	3	2.75	E65E
G252A	G	G	G	1	0.92	Q84Q
C256T	C	C	C	1	0.92	L86L
T288C	T	T	T	1	0.92	S96S
G299A	A	G	G	5	4.59	G100E
A351C	A	A	A	1	0.92	K117N
T377C	T	T	T	1	0.92	L126S
T379C	T	T	T	1	0.92	F127L
T519C	T	T	T	1	0.92	S173S
A531T	A	A	A	1	0.92	G177G
T557C	T	T	T	31	28.44	I186T
C568T	C	C	C	1	0.92	P190S
T583C	T	T	T	1	0.92	L195L
T600C	T	T	T	2	1.83	T200T
G651A	G	A	G	62	56.88	V217V
T658A	A	T	T	4	3.67	S220T
T723C	C	T	T	3	2.75	F241F
T779C	T	T	T	2	1.83	L260S
T784A	T	T	T	1	0.92	L262T
A844T	A	A	A	1	0.92	K282*
T939C	T	T	T	1	0.92	R313R
A945G	A	A	A	1	0.92	T315T
A969G	A	A	A	1	0.92	K323K
A978G	G	G	G	35	32.11	I326M
A1068C	A	A	A	8	7.34	E356D
C1177T	T	C	T	1	0.92	L393L
A1294C	A	A	A	5	4.59	R432R
T1369C	T	T	T	5	4.59	L457L
T1374G	T	T	T	1	0.92	T458T
A1380G	A	A	A	1	0.92	L460L
T1390C	C	T	T	15	13.76	L464L
T1437C	T	T	T	2	1.83	A479A
G1473A	G	A	G	30	27.52	M491I
T1479C	C	T	T	1	0.92	F493F
C1480T	T	C	T	28	25.69	L494L
T1512G	T	T	T	2	1.83	S504S
T1528C	T	T	T	1	0.92	L510L
C1539T	C	C	C	1	0.92	Y531Y
A1683G	A	A	A	1	0.92	P561P
T1687C	T	T	T	1	0.92	L563L
C1721T	C	C	C	1	0.92	S574F
T1731G	T	T	T	3	2.75	P577P
T1732A	T	T	T	1	0.92	Y578N
C1901T	C	C	C	1	0.92	S634F

AA: Asian–American lineage, As: Asian lineage, Af: African lineage

**Table 3:** HPV16 E2 gene variations and amino acid substitutions

HPV16 E2 nucleotide variation							
Variant site	AA	As	Af	Number of variation samples	Variation frequency	Amino acid change	Structural domain
				(n = 91)	(%)		
C1901T	C	C	C	1	1.10	L4F	transcriptional activation domain
G1964A	G	A	G	27	29.67	D25N	
A2074G	A	A	A	2	2.20	T61T	
A2091C	A	A	A	1	1.10	N67T	
G2129A	G	G	G	2	2.20	E80K	
G2204A	G	G	G	5	5.49	A105T	
C2295A	A	A	A	34	37.36	T135K	
G2315A	G	G	G	1	1.10	E142D	
T2341C	T	T	T	2	2.20	G150G	
G2369T	G	G	G	1	1.10	V160F	
G2385A	A	A	A	29	31.87	R165Q	
T2410G	T	T	T	13	14.29	D173E	
G2504A	G	G	G	1	1.10	V205I	Hinge region
C2511T	C	C	C	1	1.10	S207F	
T2520C	T	C	T	22	24.18	I210T	
C2546T/A	T	T	T	70	76.92	P219S/T	
G2585A	A	A	A	19	20.88	E232K	
A2587C	A	A	A	1	1.10	E232K	
T2660C	T	C	T	17	18.68	L257L	
T2703G	T	T	T	1	1.10	F271V	
C2820A	A	A	A	25	27.47	T310K	DNA binding domain
C2923A	A	A	A	4	4.40	D344E	
C2934T	C	C	C	1	1.10	S348F	
T2956C	T	T	T	1	1.10	T355T	
T2983C	T	T	T	9	9.89	S364S	

AA: Asian–American lineage, As: Asian lineage, Af: African lineage

Among the 165 HPV16 positive samples, 109 samples with HPV16 E1 variation and 48 sites with nucleotide variation were included, including 19 missense variations and 29 synonymous variations. The sample numbers of the HPV16 E1 nucleotide variation sites shown in Table 2 above for more than one missense variation are T98C, A117C, A189C, G299A, T557C, T658A, T779C, A978G, A1068C, G1473A. Variation sites and mutation frequency are shown in the Table 2. The most common nucleotide variation sites for HPV16 E1 were A189C (18/109, 16.5%), T57C (31/109, 28.4%), A978G (35/109, 32.1%), and G1473A (30/109, 27.5%). Among the 109 mutated samples, 29 sites with synonymous variations were found, including nt (nucleotide)105 (T–C) (1/109), nt195 (G–A) (3/109), nt252 (G–A) (1/109), nt256 (C–T) (1/109), nt288 (T–C) (1/109), nt519 (T–C) (1/109), nt531 (A–T) (1/109), nt583 (T–C) (1/109), nt600 (T–C) (2/109), nt651 (G–A) (62/109), nt723 (T–C) (3/109), nt939 (T–C) (1/109), nt945 (A–G) (1/109), nt969 (A–G) (1/109), nt1177 (C–T) (1/109), nt1294 (A–C) (5/109), nt1369 (T–C) (5/109), nt1374 (T–G) (1/109), nt1380 (A–G) (1/109), nt1390 (T–C) (15/109), nt1437 (T–C) (2/109), nt1479 (T–C) (1/109), nt1480 (C–T) (28/109), nt1512 (T–G) (2/109), nt1528 (T–C) (1/109), nt1539 (C–T) (1/109), nt1683 (A–G) (1/109), nt1687 (T–C) (1/109), and nt1731 (T–G) (3/109). In addition, there was a 63-base insertion sequence between nt510 and 511 in 32 samples (32/109, 29.4%).

Among the 165 HPV16 positive samples, 91 samples with HPV16 E2 variation and 25 sites with nucleotide variation were selected, including 20 missense variations and five synonymous variations. The sample numbers of HPV16 E2 nucleotide variation sites shown in Table 3 above are for those with more than one missense variation in the transactivation domain of HPV16 E2: G1964A (27/91), G2129A (2/91), G2204A (5/91), C2295A (34/91), G2385A (29/91), T2410G (13/91). Amino acids were, respectively, converted from aspartic acid to asparagine (D25N), glutamic acid to lysine (E80K), alanine to threonine (A105T), threonine to lysine (T135K), arginine to glutamine (R165Q), and aspartic acid to glutamic acid (D173E). The variants of T2520C (22/91), C2546T (41/91), C2564A (29/91) and G2585A (19/91) are located in the hinge region of HPV16 E2, with amino acids converted from isoleucine to threonine (I210T), proline to serine/threonine (P219S/T), and glutamic acid to lysine (E232K), respectively. The variants of C2820A (25/91) and C2923A (4/91) are located in the DNA binding domain of HPV16 E2, with the amino acids converted from threonine to lysine (T310K) and aspartic acid to glutamic acid (D344E), respectively. One sample each had variations at C1901T, A2091C, G2315A, G2369T, G2504A, C2511T, A2587C, T2703G, and C2934T. The amino acid changes were leucine to phenylalanine (L4F), aspartic acid to threonine (N67T), glutamic acid to aspartic acid (E142D), valine to phenylalanine (V160F), valine to isoleucine (V205I), serine to phenylalanine (S207F), glutamic acid to lysine (E232K), phenylalanine to valine (F271V), and serine to phenylalanine (S348F). The most common nucleotide variation sites for HPV16 E2 were G196A (27/91, 29.7%), C2295A (34/91, 37.4%), G2385A (29/91, 31.9%), C2546T (41/91, 45.1%), and C2564A (29/91, 31.9%). Among the 91 mutated samples of HPV16 E2, the sites of synonymous variation were NT 2074 (A–G) (2/91), NT 2341 (T–C) (2/91), NT 2660 (T–C) (17/91), NT 2956 (T–C) (1/91) and NT 2983 (T–C) (9/91).

### Phylogenetic tree analysis of nucleotide sequence of HPV16 E1 and E2

**Fig. 1:**
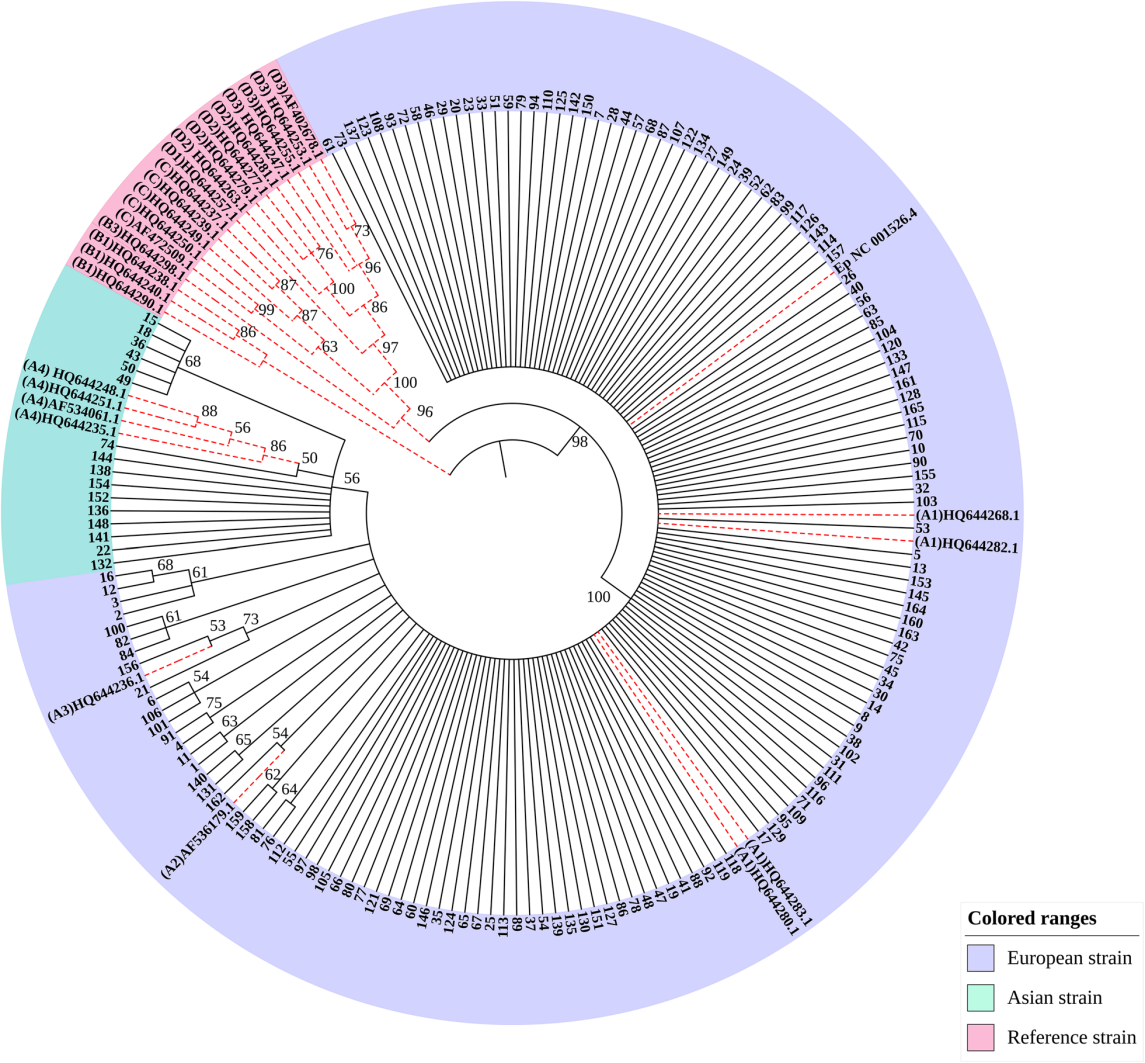
Phylogenetic tree analysis of HPV16 E1 and E2 genes

The phylogenetic tree was constructed using the N-J (neighbor-joining) method, the bootstrap method (1000 replications), and the Kimura two-parameter model. A bootstrap value > 50% indicates credibility, and a bootstrap value > 70% indicates high credibility. The nodes with bootstrap value < 50% were hidden in the evolutionary tree diagram in Fig. 1. From the phylogenetic tree results, 149 samples were European strains and 16 were Asian strains. No African or North American/Asian strains were found. The variations in European strain samples 6, 101, and 106 were shown to be all associated with the A117C variation by constructing a phylogenetic tree analysis of the nucleotide sequence of the HPV16 variant. The variations in samples 15, 18, 36, 43, 49, and 50 were all associated with the T2410G variation and all were Asian strains.

### Genetic variation of genomic HPV16 E1 in case and control groups

Samples with pathological information were counted, including 66 cases in normal group and 50 cases in cervical cancer group. All listed in the Table 4 are misspelled variations. There were 9 variations in the normal group and 11 variations in the cancer group. The variation of two sites showed that there was significant difference between the case group and the control group (*P* < 0.05). They are A978G(*P* = 0.047) and G1473A(*P* < 0.001), The amino acid changes were I326M and M491I, respectively. The E1 protein is a viral replication protein conserved in papillomaviruses, and plays roles in inducing the DNA damage response and disturbing the normal cell cycle, depending on its DNA binding and ATPase/helicase domains [20]. The difference between the case group and the control group can further explain that the variations at these two sites may promote the expression of replication protein E1, thus reducing the host cell defense capacity, and finally leading to the occurrence and development of cervical cancer.

**Table 4:** Genetic variation of genomic HPV16 E1 in case and control groups

Structural domain	Control groups (n = 66)		Case (n = 50)		*P*-values	Amino acid change
	Number of variation samples	Variation frequency (%)	Number of variation samples	Variation frequency (%)		
T98C	0	0.0	2	4.0	0.101	I33T
A117C	1	1.5	0	0.0	0.382	E39D
A189C	3	4.5	7	14.0	0.072	E63D
G299A	1	1.5	0	0.0	0.382	G100E
T557C	6	9.1	8	16.0	0.258	I186T
T779C	1	1.5	0	0.0	0.382	L260S
A978G	14	21.2	19	38.0	0.047	I326M
A1030G	0	0.0	1	2.0	0.249	T344A
A1068C	3	4.5	6	12.0	0.137	E356D
G1171T	0	0.0	1	2.0	0.249	A391S
G1473A	7	10.6	22	44.0	< 0.001	M491I
C568T	0	0.0	1	2.0	0.249	P190S
A844T	0	0.0	1	2.0	0.249	K282*
C1901G	0	0.0	1	2.0	0.249	S634C
C1907A	1	1.5	0	0.0	0.382	P636Q

### Genetic variation of genomic HPV16 E2 in case and control groups

Table 5 shows the HPV16 E2 gene variation in normal group and cervical cancer group. All listed in the Table 5 are missense variations. There were 13 variations in the normal group and 16 variations in the cancer group. The variation of some sites showed that there was significant difference between the case group and the control group (*P* < 0.05). HPV16 E2 variations located at site G1964A (*P* = 0.017), C2295A (*P* = 0.009) and G2385A (*P* = 0.004) in the transactivation domain, The amino acid changes were D25N, T135K and R165Q, respectively. The sites T2520C (*P* = 0.002), C2546T (*P* < 0.001) and G2585A (*P* = 0.009) in hinge region, with corresponding amino acid changes of I210T, P219S and E232K, respectively. The sites C2820A (*P* = 0.002), C2873T (*P* = 0.044) and C2923A (*P* = 0.001) in DNA binding domain, the amino acid changes were T310K, H328Y and D344E. Since the E1 protein binds to the trans-activation domain of the E2 protein and co-localizes at the start site of transcription, it co-regulates viral transcription [21, 22], The variation of hinge region and DNA binding domain will affect the binding of E2 protein to LCR and inhibit the expression of E6 and E7 protein [23].

**Table 5:** Genetic variation of genomic HPV16 E2 in case and control groups

Structural domain	Variant site	Control groups (n = 66)		Case (n = 50)		*P*-values	Amino acid change
		Number of variation samples	Variation frequency (%)	Number of variation samples	Variation frequency (%)		
Transcriptional activation domain	G1964A	10	15.2	17	34.0	0.017	D25N
	G2204A	2	3.0	2	4.0	0.777	A105T
	C2295A	11	16.7	19	38.0	0.009	T135K
	G2315A	1	1.5	0	0.0	0.382	E142D
	G2385A	7	10.6	16	32.0	0.004	R165Q
	T2394A	0	0.0	1	2.0	0.249	I168Y
	T2410G	5	7.6	7	14.0	0.261	D173E
Hinge region	T2520C	7	10.6	17	34.0	0.002	I210T
	C2546T	17	25.8	36	72.0	< 0.001	P219S
	G2585A	8	12.1	16	32.0	0.009	E232K
	G2621C	1	1.5	0	0.0	0.382	E244Q
	A2636C	0	0.0	1	2.0	0.249	N249H
	T2672C	3	4.5	4	8.0	0.439	S261P
	T2703G	0	0.0	2	4.0	0.101	F271V
DNA binding domain	C2820A	8	12.1	18	36.0	0.002	T310K
	C2873T	0	0.0	3	6.0	0.044	H328Y
	C2923A	1	1.5	10	20.0	0.001	D344E
	C2934G	0	0.0	2	4.0	0.101	S348C

## Discussion

Some nucleotide variations in HPV16 affect amino acid changes and may also affect protein expression, thus, affecting the development of cervical cancer. Most studies on HPV nucleotide polymorphism have focused on E6 and E7, and variation of E6 and E7 can affect the occurrence and development of cervical cancer [7]. Variations that destroy the ORF(open reading frame) of HPV16 E1 or E2 lead to a further increase in the immortalization potential of human keratinocytes [16]. There is a 63-base insertion sequence between nt510 and 511 of HPV16 E1, and this mutant is associated with viral oncogenic activity and viral integration [24]. Variation of T310K in the HPV16 E2 DNA binding domain may alter cellular transcription factors, where the E2 protein interacts with LCR, resulting in enhanced expression of E6 and E7 proteins [25]. E232K is a linked variation in the E2 hinge region of HPV 16 AS that enhances dose-dependent inhibition of LCR (Long control region) activity and may affect the potential of the virus to cause cancer [26].

Of the 165 samples with HPV16 infection analyzed in Xinjiang, 149 samples were European strains and 16 samples were Asian strains; no African or North American/Asian strains were found. Nucleotide variation of HPV16 E1 occurred in 109 samples with 48 variation sites, and the amino acids of 19 samples were changed. The most common nucleotide variation sites were A189C (18/109, 16.5%), T57C (31/109, 28.4%), A978G (35/109, 32.1%), and G1473A (30/109, 27.5%). The amino acid changes were E63D, I186T, I326M, and M491I, respectively. Sequence analysis of HPV16 E2 revealed that nucleotide variations occurred in 91 samples with 25 variation sites, of which 20 sites caused amino acid changes. The most common nucleotide variation sites of HPV16 E2 were G1964A (27/91, 29.7%), C2295A (34/91, 37.4%), G2385A (29/91, 31.9%), C2546T (41/91, 45.1%), and C2564A (29/91, 31.9%), with corresponding amino acid changes of D25N, T135K, R165Q and P219S/T, respectively. Two variants, C–T and C–A, occur in the nucleotide sequence nt2545. The variation in three samples was associated with the A117C (E39D) variation and in six samples was associated with the T2410G (D173E) variation. The nucleotide variations at sites G195A (3/109, 2.75%), G252A (1/109, 0.92%), A351C (1/109, 0.92%), T377C (1/109, 0.92%), T379C (1/109, 0.92%), T519C (1/109, 0.92%), C568T (1/109, 0.92%), T600C (2/109, 1.83%), T784A (1/109, 0.92%), A844T (1/109, 0.92%), T939C (1/109, 0.92%), A945G (1/109, 0.92%), A969G (1/109, 0.92%), and A1380G (1/109, 0.92%) have not been reported. HPV16 E2 variations located at site A2091C (1/91,1.10%) in the transactivation domain, G2129A (2/91,2.20%), G2204A (5/91,5.49%), G2369T (1/91,1.10%), A2587C (1/91, 1.10%), and T2956C in the DNA-binding domain (1/91, 1.10%) have not been reported, and there may be synergistic variations in G1964A, C2295A, G2385A, T2410G, T2520C, C2546T/A, G2585A, T2660C, and C2820A. The variation of some sites showed that there was significant difference between the case group and the control group (*P* < 0.05).In our research, the variation of two sites in E1 showed that there was significant difference between the case group and the control group (*P* < 0.05). They are A978G (*P* = 0.047) and G1473A (*P* < 0.001), The amino acid changes were I326M and M491I, respectively. HPV16 E2 variations located at site G1964A (*P* = 0.017), C2295A (*P* = 0.009) and G2385A (*P* = 0.004) in the transactivation domain, The amino acid changes were D25N, T135K and R165Q, respectively. The sites T2520C (*P* = 0.002), C2546T (*P* < 0.001) and G2585A (*P* = 0.009) in hinge region, with corresponding amino acid changes of I210T, P219S and E232K, respectively. The sites C2820A (*P* = 0.002), C2873T (*P* = 0.044) and C2923A (*P* = 0.001) in DNA binding domain, the amino acid changes were T310K, H328Y and D344E.These base variations will lead to amino acid variations, which may affect the expression of proteins. In the future, we will study the role of HPV16E1 and E2 variants in the occurrence and development of cervical cancer based on experiments studying cell function.

## Conclusion

From the phylogenetic tree results, 149 samples were of the European variant and 16 samples were of Asian variant type. No African or North American/Asian variant types were found.

## Materials and methods

### Collection of samples

A total of 165 HPV16 positive samples were collected from exfoliated cervical cells of women with cervical lesions at Yili Friendship Hospital, Kashi People's Hospital and Xinjiang Uyghur Autonomous Region People's Hospital; informed consent was obtained from all patients. None of the patients had a history of long-term residence abroad. The samples were stored at − 80 °C.

### DNA extraction and PCR amplification of samples

DNA extraction was carried out using a genomic DNA extraction kit (Shanghai Sangon Bio-logical Engineering Technology and Services Company, Shanghai, China), and the extracted DNA was stored at – 20 °C. A 1 µl DNA sample was mass examined and its concentration estimated by 1% agarose electrophoresis; it was then diluted to a working concentration of 10–20 ng/µl. Samples without significant DNA bands were not diluted. The reaction mixture (20 µl) included 1 × HotStarTaq buffer, 2.0 mM Mg^2+^, 0.2 mM dNTP, 0.2 µM of each primer, 1 U HotStarTaq polymerase (Qiagen Inc.) and 1 µl template DNA. The cycling program was 95 °C for 5 min; 30 cycles × (94 °C for 45 s, 50 °C for 45 s, 7 °C for 1 min); 72 °C for 5 min; 4 °C to finish. The PCR product could be stored in a refrigerator at 4 °C. PCR primer information is shown in Table 1.

### Sequencing

Sequencing was performed by Shanghai Tianhao Technology Co., Ltd., and the PCR product was purified by SAP (Promega) and EXO I (Epicentre): 0.5 U SAP and 4 U Exo I were added to 8 µl PCR products. The mixture was incubated at 37 °C for 60 min, followed by incubation at 75 °C for 15 min. Finally, the BIG-DYE Terminator V3.1 cycle sequencing kit from ABI Co. was used for sample sequencing on a DNA analyzer (ABI3130XL) after purification with alcohol. The sequencing primers were: E-1R: 5′-ACTGCAACCACCCCCACT-3′, E-2R: 5′-ATCCATTCTGGCGTGTCTCC-3′, E-3F: 5′-TCATGGGGAATGGTTGTGTT-3′, E-4F: 5′-GGTGATTGGAAGCAAATTGTTATG-3′, E-5R: 5′-CCCGCATGAACTTCCCATAC-3′, E-6F: 5′-GAAGCATCAGTAACTGTGGTAGAGG-3′, as shown in Table 1.

### Phylogenetic analysis of HPV16 variants

The sequencing results were analyzed for single nucleotide polymorphisms (SNPs) using the Polyphred software, compared to the European standard prototype (GenBank: NC_001526.2) and compared to other typical HPV variants: HQ644283.1 (A1), HQ644268.1 (A1), HQ644280.1 (A1), HQ644282.1 (A1), AF536179.1 (A2), HQ644236.1 (A3), HQ644248.1 (A4), HQ644251.1 (A4), AF534061.1 (A4), HQ644235.1 (A4), HQ644240.1 (B1), HQ644290.1 (B1), HQ644238.1 (B1), HQ644298.1 (B3), HQ644237.1 (C), HQ644239.1 (C), HQ644249.1 (C), HQ644250.1 (C), AF472509.1 (C), HQ644257.1 (D1), HQ644279.1 (D2), HQ644281.1 (D2), HQ644263.1 (D2), HQ644277.1 (D2), HQ644247.1 (D3), HQ644253.1 (D3), HQ644255.1 (D3), AF402678.1 (D3). The evolutionary tree was constructed using MEGA7.

### Statistical analysis

The frequency of each mutation in the HPV16 E1 and E2 genes was determined by direct enumeration. A chi-square test was performed to determine the association between HPV16 E1, E2 variants and cervical cancer. Statistical analysis was performed using SPSS 17. *P*-values < 0.05 were considered statistically significant.

## Data Availability

The datasets generated and/or analysed during the current study are available in the NCBI GenBank repository,GenBankID:MZ151424/MZ151425.[link:https://www.ncbi.nlm.nih.gov/nuccore/MZ151424, https://www.ncbi.nlm.nih.gov/nuccore/MZ151425].
